# Convolutional Neural Networks Approach for Solar Reconstruction in SCAO Configurations

**DOI:** 10.3390/s19102233

**Published:** 2019-05-14

**Authors:** Sergio Luis Suárez Gómez, Carlos González-Gutiérrez, Francisco García Riesgo, Maria Luisa Sánchez Rodríguez, Francisco Javier Iglesias Rodríguez, Jesús Daniel Santos

**Affiliations:** 1 Department of Mathematics, University of Oviedo, Calvo Sotelo s/n, 33007 Oviedo, Spain; suarezsergio@uniovi.es; 2 Department of Computer Science, University of Oviedo, Campus of Viesques s/n. 33024 Gijón, Spain; gonzalezgcarlos@uniovi.es; 3 Department of Physics, University of Oviedo, Calvo Sotelo s/n, 33007 Oviedo, Spain; uo240058@uniovi.es (F.G.R.); mlsr@uniovi.es (M.L.S.R.); 4 Department of Business Administration, University of Oviedo, Avda. del Cristo s/n, 33006 Oviedo, Spain; sanchezfernando@uniovi.es

**Keywords:** artificial neural networks, convolutional neural networks, adaptive optics, solar observations, solar adaptive optics

## Abstract

Correcting atmospheric turbulence effects in light with Adaptive Optics is necessary, since it produces aberrations in the wavefront of astronomical objects observed with telescopes from Earth. These corrections are performed classically with reconstruction algorithms; between them, neural networks showed good results. In the context of solar observation, the usage of Adaptive Optics on solar differs from nocturnal operations, bringing up a challenge to correct the image aberrations. In this work, a convolutional approach is given to address this issue, considering SCAO configurations. A reconstruction algorithm is presented, “Shack-Hartmann reconstruction with deep learning on solar–prototype” (proto-HELIOS), to correct on fixed solar images, achieving an average 85.39% of precision in the reconstruction. Additionally, results encourage to continue working with these techniques to achieve a reconstruction technique for all the regions of the sun.

## 1. Introduction

One of the biggest challenges to obtain diffraction limited images in grounded telescopes is the correction of the effect that the atmospheric turbulence produces on the incoming light from astronomical sources [1].

Incoming star light is refracted in different ways due to the existence in the atmosphere of areas with higher and lower air density, which have a different refractive index and are moved around by random winds. A way of avoiding the disturbing effect of atmosphere is the use of Adaptive Optics (AO) to correct the turbulence that induces distortions in the incoming wavelengths. AO has been developed in the last decades to improve night astronomy and aims to obtain near diffraction-limited images.

AO is an example of how new technological tools and techniques support the development of science, such as Astronomy and Astrophysics. Basically, AO measures the atmospheric turbulence effects, reconstructs the incoming Wavefront, calculates a correction and employs a flexible active mirror that reverses the distortions caused by the atmosphere [2].

However, the use of AO is not restrained to nocturnal observations. Research on processes that take place in the sun have an enormous interest, since it strongly affects life on Earth [3]. The extremely dynamic behavior of the solar atmosphere influences the Earth. The change of the sun luminosity affects climate on Earth, and the behavior of the sun chromosphere electrodynamics is closely related to the sun’s magnetic field.

The study of the phenomena taking place on the sun’s atmosphere would lead to the understanding of the solar magnetic field, its self-induced dynamo mechanism and its relationship to the ejection of solar material from the solar corona that reaches the Earth as solar wind, and strongly affects the Earth’s magnetosphere.

This leads to assess the solar observation for its improvement with the use of AO in solar telescopes, with similar techniques as in night AO and the help provided by reconstruction systems. Nevertheless, solar AO presents some important differences with respect to night observation that must be considered for the adequate working of AO systems.

Typically, nocturnal AO, of which the most widely employed sensor is the Shack-Hartmann Wavefront sensor (SH WFS), consists of an array of lenses that divide the wavefront into discrete areas [4]. However, there are more types of WFS that are used in nighttime observations, such as the curvature sensor [5], which measures directly the Laplacian of the wavefront surface at the aperture edge in a perpendicular direction to the edge. The plenoptic sensor [6], which has a similar structure to the SH WFS, is based on an array of lenses, but its cameras and reconstruction algorithm avoid the ambiguities caused by low intensity interferences. In this sensor, the array of lenses is located at the telescope focus instead of the pupil plane like SH one. Another possible sensor is the pyramid sensor [7], which is characterized by having a pyramid shape prism that divides the outgoing beam of each lens into new four beams in different directions.

The main goal of this work is to affront the challenge of reconstruction in solar AO. In particular, the presented approach relies in a specific type of sensor, that allows to perform measurements of the atmospheric distortions for diurnal observations, the Cross-Correlation Shack-Hartmann [8]. This sensor is the standard used in solar, being a modification of the classical SH, that provides the adequate information for a posterior phase recovering by means of a reconstruction technique; in solar telescopes imaging a point source is not available being, instead, an extended scene. Sensors would form, in the lenslet array, an array of subimages [9]; the shifts between the images allows to estimate the turbulence effect, which in nocturnal AO was estimated directly from the centroids, by means of the computation of the cross-correlations [10]. Due to the slight practical experience with other sensors in solar AO, the majority of telescopes are set to use Cross-Correlation SH, as the European Solar Telescope (EST) [11].

Diurnal observations are very conditioned by the energy that is conferred by the Sun to the atmosphere. It makes stronger turbulences with greater capacity of change. This is supported along with the necessity of the development of solar telescopes, as the Swedish Solar Telescope (SST), a 1 m diameter telescope located in La Palma [12,13] for solar observation that has an integrated AO system characterized by having two bimorphic deformable mirror (DM) [14], which produce fewer aberrations than those formed by discrete actuators but the capacity they have to adapt to different forms is inferior, or the EST [15], a 4 m telescope designed to be located in the Canary Islands, capable to be adapted to a wide range of AO configurations [16].

Measurements obtained from wavefront sensors are used, in both solar and nocturnal AO, to estimate the wavefront phase, with the use of a reconstruction algorithm. The calculated wavefront is used, through a control system, to determine the shape that a DM has to adapt to compensate the aberrations suffered by the image taken by the telescope.

The use of Artificial Neural Networks (ANNs), a mathematical model from the field of Artificial Intelligence, is widely known for the uses in pattern recognition and prediction [17,18], image [19], speech and handwriting processing, and all sorts of complex modeling of physical system [20,21]. In particular, these techniques have been proven to be successful in several scenarios for nocturnal AO [22].

For that, we present a first approximation of ANN reconstruction for solar observations, proposing as result the network “Shack-Hartmann reconstruction with deep learning on solar–prototype” (proto-HELIOS). This network successfully corrects atmospheric turbulence for static sections of the images in the sun, proving the ANN’s potential in solar AO reconstruction and also sets the first steps in the development on ANN-based reconstruction techniques with wider capacity. This first approximation with Artificial Intelligence is a response to a currently open problem, which other researchers also tried to address with more classical approaches such as the Least Squares method [23] and its adjustments [24,25].

In this work, Section 2 presents the methods required, particularly, the basics of AO and the relation and differences with solar AO, as well as the AO configuration chosen for this work; moreover, the working of neural networks is included and the last subsection, which details the experimental procedure from simulations to network determination. Section 3 presents the results obtained from the experimentation explained above, and Section 4 details and discuss the results. Finally, Section 5 summarizes the conclusions of this work.

## 2. Materials and Methods

### 2.1. Adaptive Optics

The continuous changes in the atmospheric turbulence implies that the incoming light from stellar objects found their wavefront aberrated as it passes through the atmosphere. Turbulence is usually stronger on the ground layer at the quiet sites where telescopes are located.

One of the models used to characterize the atmosphere are Kolmogorov models [26], where the turbulence can be characterized by a series of parameters as the coherence length $r_{0}$ [27], which gets smaller as turbulence gets stronger and depends on the wavelength [28], or others [29] such as turbulence layer altitudes, wind parameters, etc.

**Figure 1:**
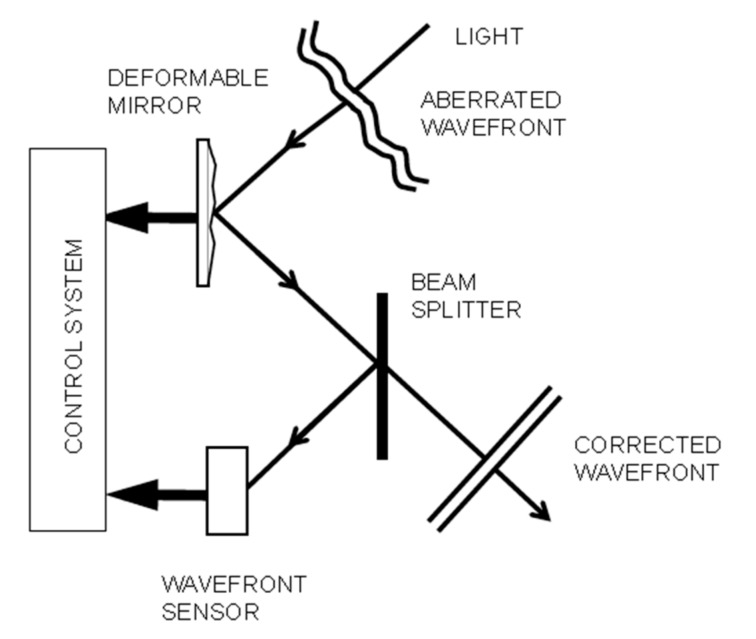
Concept of the working of an Adaptive Optics (AO) system.

The aberrated incoming light is measured by wavefront sensors (WFS); after the corrections are estimated by the reconstructor, they are sent to a deformable mirror (DM) to obtain a plane wavefront [30,31]. To perform an effective correction, the control system must execute all calculations and corrections in few milliseconds, due to the quick evolution of atmospheric turbulence. A diagram of the general working of an AO system is represented in Figure 1.

The wavefront measurements must be performed with high spatial resolution and speed to apply a real-time correction. The SH WFS provides the slopes of the wavefront to be corrected. The centers of the images are obtained with the help of algorithms that compute their gravity center, giving as result the centroids; focused points in the subapertures that characterize the slopes in the correspondent differential areas of the wavefronts.

The incoming light to be measured by WFS is provided in astronomical night observation by a guide star, that could be a Natural Guide Star near the observation objective, or an artificial Laser Guide Star [32]. The correction is made for the plane perpendicular to the direction of the light, but the turbulence scale is much smaller. One part of the atmosphere distorts the beam in a different way than another part. This is called anisoplanatism and is a major problem for the case of large telescope apertures [33].

**Figure 2:**
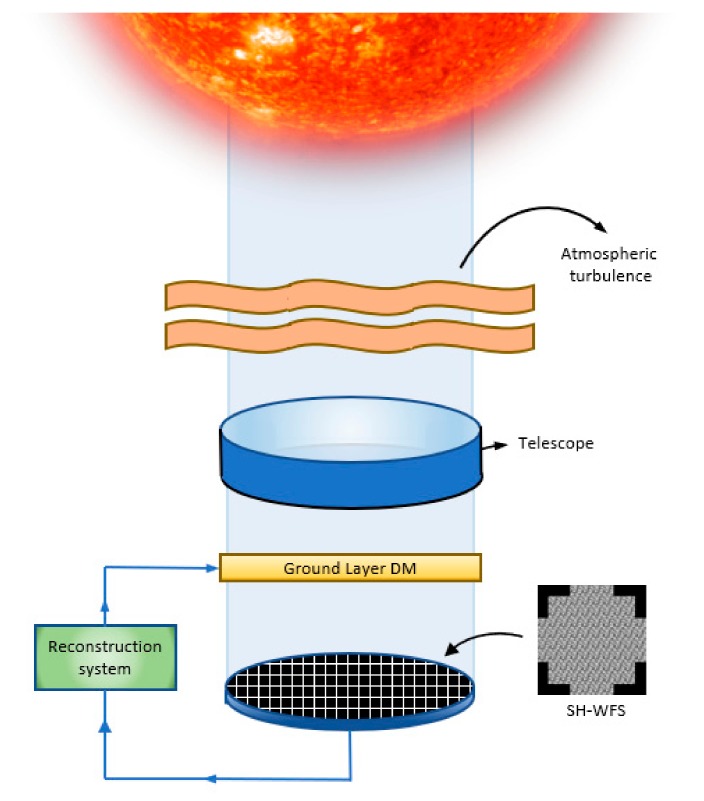
Solar Single Conjugate Adaptive Optics (SCAO) system.

The basic and simplest configuration for AO purposes is the Single Conjugate Adaptive Optics (SCAO), which has been first tried to develop our control system for solar observation [34]. Figure 2 shows a sketch of the Solar SCAO system, with only one wavefront sensor and only one conjugated deformable mirror.

The SCAO configuration works on closed loop mode. The measured centroids are used as input for the reconstructor to calculate the received wavefront phase and the correction that must be fed to the DM actuators.

### 2.2. Adaptive Optics for Solar Observations

The diurnal observation is greatly conditioned by the fact that the energy of the Sun affects the atmosphere directly [35]. This confers greater energy to the turbulent layers, generating stronger turbulences, with the corresponding difficulty to perform observations. The control systems and reconstruction algorithms suffer from this increased difficulty, since the reconstruction carried out for a specific moment can differ much from the necessary in the following instant, which in night observation occurs slower, as the turbulences are more stable. The layer most affected by this phenomenon is the Ground Layer, also due to the heat irradiated by the earth and the pupil of the telescope during the daytime hours [36].

Although the Sun is a medium-sized star, its proximity to Earth means that it cannot be considered as a point source, since it will take up all the field of view (FOV) of each of the openings of a standard Shack-Hartmann sensor due to the large extension of the image. The measurement of the wavefront should be done without having guide stars, as were used in nighttime observation [37]. Nor are there bright enough light sources to use them as guide stars that could be detected as point sources on solar background radiation.

The first modification made when AO was applied to solar observation was the change in wavefront sensor, since SH WFS is used when there is a high contrast difference in the image, like in night observation. Since the sun is a widefield object, the use of traditional SH to perform wavefront measures implies that the sensor’s lenslets would be filled, not allowing the proper calculation of the centroids; consequently, an alternative is needed to perform measurements that estimates the atmospheric turbulence in diurnal situations. The use the Cross-Correlation SH allows to solve these issues [9]. The main difficulty is the absence of guide stars that allows to know the turbulence at each moment, where several frames of the sun can be used as reference instead [38]. The images produced by the lenslets of the SH WFS would be displaced from each other. One of the images of the lenslets is chosen randomly as reference image, in order to compare it to all the images of the subapertures, the cross-correlation in real time (CC in real time) is calculated, using a traditional method [39] for its simplicity, although other more recent methods also offer good results [9,40]. This traditional approach needs a smaller number of calculations, shorter calculation times and less powerful equipment, which is enough for a first trial to explore the development of our solar AO reconstructor.

From the images collected in the Shack-Hartmann sensor, the aberration suffered by the wavefront is calculated. The aberration will be calculated by computing cross correlations (CC).

During the initial stage, a reference pattern of the area to be served is generated, such as solar granulation. During the following steps, successive images of the same area are obtained and the displacement with respect to the reference image is calculated. The full cross-correlation functions of the live images are calculated by fast Fourier transforms.

An initial image of the area is taken, which will be taken as a reference. Any image can be taken, but generally one of the brightest should be chosen. From it, the cross-correlation between the live image taken at each moment and the reference image is calculated according to the following equation [39]:

(1) $$CC_{SR}\left(\Delta \right)=\int \int I_{S}\left(x\right)\times I_{R}\left(x+\Delta \right)dx$$

where $I_{S}\left(x\right)$ is the live image that is obtained at each instant and $I_{R}\left(x\right)$ is the image taken initially as the reference image, where x denotes the spatial coordinate on the sensor undershoot and Δ denotes a spatial delay, the result of obtaining the image in a different subaperture.

The covariance is computed by making a Fourier transform [41]:

(2) $$CC_{SR}\left(\Delta \right)=F^{+}\left[F^{-}[I_{S}\left(x\right)\right]\times F^{-*}\left[I_{R}\left(x\right)\right]]$$

where $F^{-}$ and $F^{+}$ the direct and inverse Fourier transform, respectively. $F^{-*}$ denotes the complex conjugate of the Fourier Transform.

The position of the maximum of the cross-correlation CC is a linear measure of the displacement between the reference image and the given subaperture, being an estimation that represents the atmospheric turbulence, which will be carried out in the same way as it was done in the nocturnal adaptive optics, starting from the centroids of each subaperture.

Once these values are calculated, the wavefront received can be recovered by a reconstructor, in terms of Zernike polynomials [42], DM actuator vales, etc., depending on the reconstruction algorithm implemented. This allows the control system to obtain the values of the positions that each DM section must occupy for the correction of the aberration suffered.

### 2.3. Artificial Neural Networks

Neural networks are one of the main algorithms in Artificial Intelligence nowadays. The mathematical model of an ANN is developed to mimic the process of interconnections and learning of a biological neural system. The purpose of the use of ANNs in AO is to work as a control system for AO configurations.

These networks are formed by individual processing units called neurons. Each neuron receives an input information signal, processes it and calculates a response or output signal [43]. Each neuron is connected with all the neurons of the following layer; these connections are characterized with weights, which is interpreted as the influence that the preceding neuron will have on the result of the current one.

When the structure of layers includes an input layer, where the information is gathered, one or several hidden layers, where the processing of the network is performed and an output layer that gives the response of the network, this topology in named as Multi-Layer Percepton (MLP). Usually, the response of each layer is modulated by an activation function [33].

**Figure 3:**
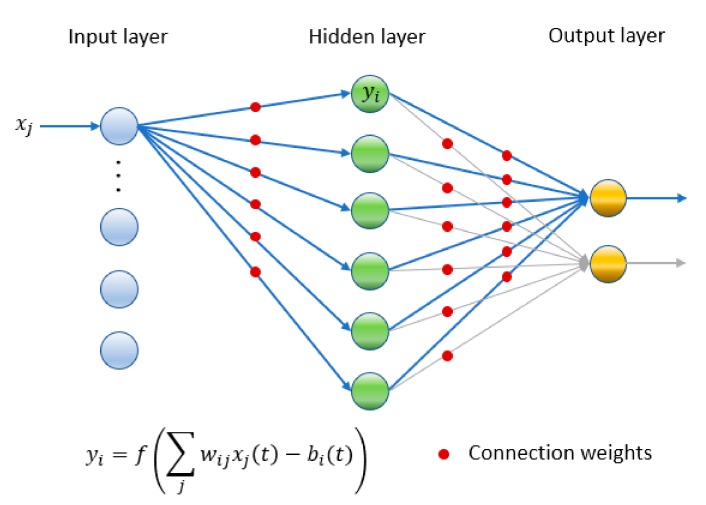
Example of Multi-Layer Perceptron topology, where connections for neurons of consecutive layers are characterized by weights. The output of each layer is produced by the application of an activation function in the lineal combination of the inputs and the weights. The output is obtained with the response given by the output layer, after the sequences of hidden layers.

As it is shown in the diagram of an MLP topology in Figure 3, the working of the networks is as follows:

- Input set $x_{j}$.
- Synaptic weights $w_{ij}$; indicate the intensity of interaction with the neuron j the weight that the received/given information will have.
- Activation function $f\left(\sum_{j}w_{ij}x_{j}\left(t\right)-b_{i}\left(t\right)\right)$ corresponds to the final output of the neuron; including the bias.

In a convolutional neural network, the model is intended to allow the use of images, or high-dimensional data samples, that represent a computational problem when the dimension is lowered to vector shape. If, for example, an image is reshaped to a vector to be used as the inputs of a MLP network, some relevant information, as spatial position, may be lost [44]. The convolutional networks were developed to avoid this issue, using the whole sample.

A convolutional layer consists of a series of kernels; matrixes to be applied in sections of the original samples, giving as a result the sum of the elements as a Hadamard product [45] between the kernel and the image section. These kernels are intended to work as feature filters, to extract the most relevant characteristics from the sample. The kernels are applied at certain stride, requiring in some situations to add a padding to the sample. Once the kernels have been applied over all the sample, a feature map is obtained for each kernel in the convolutional layer [46].

Usually, after the layer, several post-processing can be applied before the connection with other convolutional layers. The use of an activation function, such as the Rectified Linear Unit (ReLU), Parametric Rectified Linear Unit (PReLU) or Leaky-ReLU [47] modulate the data, and the use of pooling procedures allows to select the most relevant data, reducing the size of the feature maps, for example with Max-Pooling [48].

**Figure 4:**
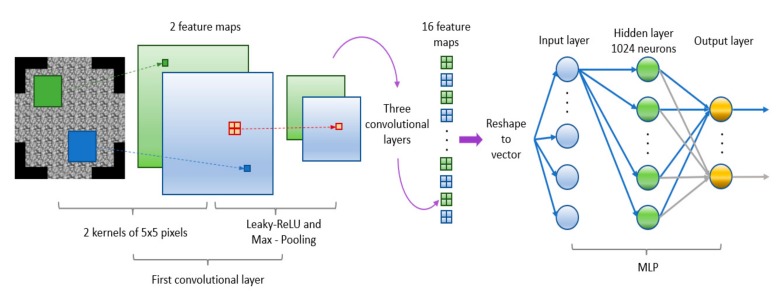
Topology and implementation of Shack-Hartmann reconstruction with deep learning on solar–prototype (proto-HELIOS). The architecture consists of four sets of convolutional layers of two kernels each, followed by Leaky-ReLU and Max-Pooling. After the convolutional process, output feature maps are reshaped to a vector and connected to a Multi-Layer Percepton (MLP).

After the convolutional processing the feature maps are connected as inputs to the input layer of the MLP section of the network topology, to finally reach an output. A diagram of the topology of a convolutional network, in particular the topology and implementation of proto-HELIOS, can be found in Figure 4.

Once a model of ANN gives an output, it is required that the network adopts the information of the complex system where it is intended to be used. This process is known as training or learning procedure and requires a training set that should be as representative as possible. The learning process consists of adjusting the weights and kernels to represent more adequately the problem.

Using input data with the known output values, the error is calculated with a loss function, usually those as Root Mean Squared Error (RMSE) or Mean Absolute Error (MAE) [49]. The error in the output is propagated back to adjust the weight and kernel values following a minimization algorithm, as gradient descent algorithm [50] or versions as Nesterov or Adagrad [51]. The backpropagation of the error is done for iterations or epochs over the full set of samples.

### 2.4. Simulation Setup

**Figure 5:**
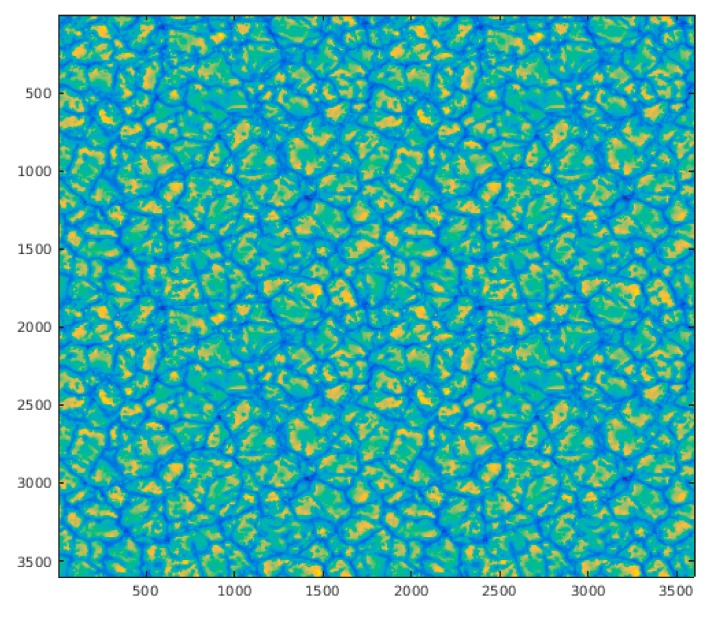
Full solar image used for simulation in DASP. The simulated telescope uses a determined frame of this image as scientific target.

**Figure 6:**
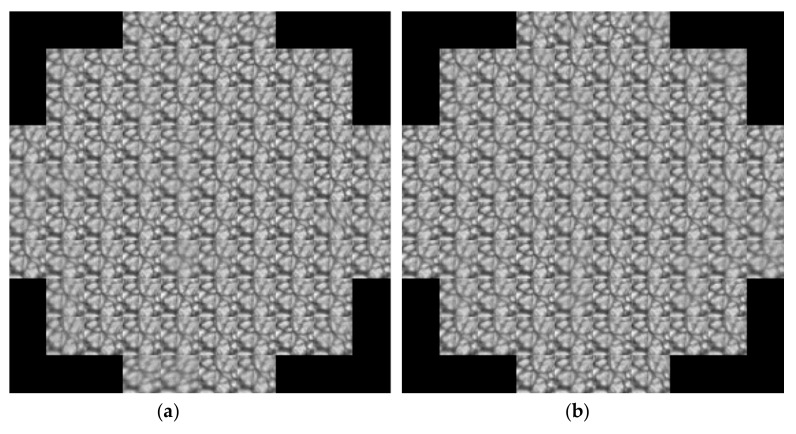
Examples of two SH measures for the same frame of the solar image, with turbulences at: (**a**) 2 km (left) and (**b**) 7.5 km (right).

The present work focuses on the estimation of DM actuator values, from SH solar images. The data has been obtained using the solar module computed in Durham Adaptive Optics Simulation Platform (DASP) [52]. The simulator uses a solar image (Figure 5) and calculates the effect of the turbulence applied to sections or frames of the image, as can be seen in the simulated SH images (Figure 6).

The development of the neural networks in this work was performed in TensorFlow [53], where the SH images were used as inputs to estimate the correspondent DM actuator values for the SCAO simulated scenario.

As described below, this work includes different approaches.

#### 2.4.1. Several Solar frames.

The network was trained to obtain DM values from the SH image considering different frames of the solar image.

- Simulations.
  During the simulations, several offsets were established to select the different frames of the solar image; eight values for pixels in the coordinates and 10 values for the pixels on the ordinates, giving a result of 80 different combinations. These conformed the regions of the sun to be trained with.
  The simulated SH had 10 subapertures per side, with a resolution of 28 pixels of side per subaperture. The simulated outputs corresponded with the 117 active DM actuators.
  For the training set, a fixed value of 0.12 cm for the $r_{0}$, taking as example the performance of other ANNs in nocturnal AO when trained [54,55]. The height steps of the turbulence were 1 km each, from 0 to 15 km of altitude. Each combination of all the above information was repeated for 100 iterations each, giving a total of 128,000 training samples.
- Network.
  The topology of the network was selected after a grid search on its hyperparameters; considering different number and sizes of kernels, as well as number of neurons on the hidden layer. Only one hidden layer was used to minimize the vanishing gradient influence. In particular, the topology consisted on four convolutional layers, with 8, 2, 2 and 2 kernels, respectively. The size of those kernels was of 5 × 5 pixels, with stride steps of 1 in both directions; additionally, padding was added. All the convolutional layers used Leaky-ReLU as activation function and were followed by max-pooling of 2 × 2 pixels for the first three layers and 5 × 5 pixels for the last one. As the image from each sample was square of 280 pixels of side, the resulting 64 images of 7 pixels of side were reshaped to a vector of 3136 components to be used as inputs of the MLP section of the network. A hidden layer was set with 1024 neurons, with Leaky-ReLU as activation function. The output layer is set for 117 neurons, corresponding with the actuator values, without any activation function. The network was trained with Adagrad procedure, using MSE as loss function, with learning rate of 0.001 and momentum of 0.9.

#### 2.4.2. Fixed Section of the Image

With the aim to train a network for a fixed frame of the solar image, a new simulation was performed with an established offset.

- Simulations.
  The sizes from both the SH and the DM remains the same as the previous case, with 10 subapertures per side of the simulated SH, with resolution of 28 pixels of side per subaperture, and 117 active DM actuators.
  The training set had a fixed value of 0.12 cm for the $r_{0}$, and height steps of 10 meters each, from 0 to 15 km of altitude. Each step was repeated for 100 iterations each, giving a total of 150,000 training samples. For establishing the test set, two approaches were taken.
  (1) Fixed $r_{0}$ tests. The test set were simulated for a fixed $r_{0}$ with height steps ok 1 km each. Varying the $r_{0}$ from 5 to 20 cm conformed the 16 tests set used.
  (2) Multi-Layer tests. In order to corroborate the capability of the neural network to generalize over more complicated scenarios, three tests [33] were used with four turbulent layers. The test properties are explained in the following Table 1:
- Network.
  As in the previous case, the topology of the network was set after a grid search on its hyperparameters, considering kernel number, size, number of hidden layer neurons, etc., selecting those that increased the performance of the model. The reconstructor proto-HELIOS consists of a convolutional neural network with four convolutional layers, each one with two kernels of 5 × 5 pixels each. All the layers use Leaky-ReLU as activation function, with strides of 1 for the kernels and adding padding in every convolutional layer, all the layers are followed by max-pooling of 2 × 2 pixels in the first 2 layers, with a 5 × 5 pixel pooling for the third one and a 7 × 7 pixel pooling for the last one.
  The convolutions result in 16 images of 2 × 2 pixels, which are reshaped in a vector to be used as the inputs of the MLP section of the network. The hidden layer has 1024 neurons, and the output has 117 neurons. Both the hidden and the output layer have Leaky-ReLU as activation function.
  The network was trained with Adagrad procedure, using MSE as loss function, with learning rate of 0.001 and momentum of 0.9.

## 3. Results

In this section, results from the neural networks are presented. The performance of all the techniques is presented in terms of normalized mean error over their application to test sets. Results have been separated depending on the case of the network for modeling several solar frames, or the network trained to work in a fixed section of the solar image.

### 3.1. Several Solar Frames

Testing the network over a test set of 10% the size of the training set, achieves a mean value of 27.53% of normalized mean error. These results are provided considering the mean result on a test set made with the same parameters of the training set, except for the frames chosen, where the test set has 10% the frames of the training and were chosen to not coincide with those of the training, in order to evaluate the generalization capability of the network.

The presented results are for the topology presented in the experimental setup.

### 3.2. Fixed Section of the Image

The results of the first series of test, where only the $r_{0}$ changed, show an expected behavior. The normalized mean error is higher in the scenarios with stronger turbulence, reaching errors of 31.81%, but lowers and stabilizes in on calmer atmospherics conditions, reaching a minimum of 11.2% of error.

The highest value of the error is found in the most turbulent scenario, when the atmospheric variations in the refractive index are high enough to induce strong aberrations in the light obtained from the observations; this behavior is found in *r*_0_ of 5, 6 and 7 cm. Although for 8 cm the turbulence still could be considered strong, the network begins to perform better phase recoveries, shown in the medium and weak turbulences.

**Figure 7:**
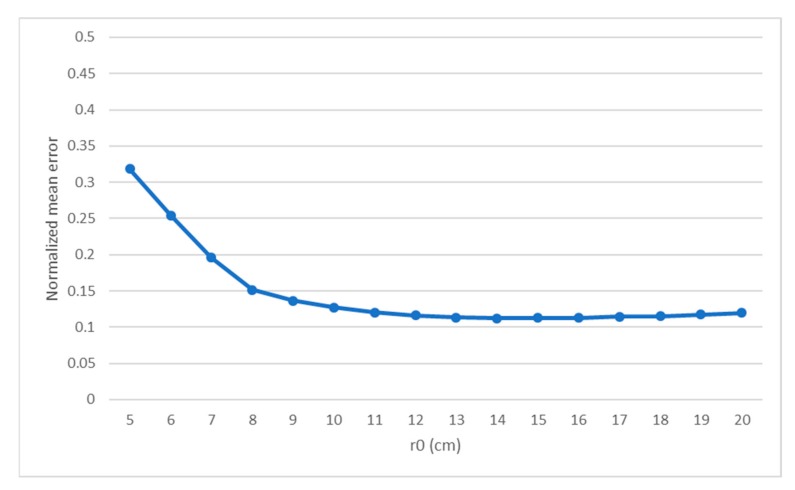
Normalized mean error for the tests varying the $r_{0}$ from 5 cm to 20 cm.

The test over different $r_{0}$ values gives the normalized mean error that can be seen in Figure 7. On average, the reconstructor has a 14.61% of error over the objective DM values.

**Figure 8:**
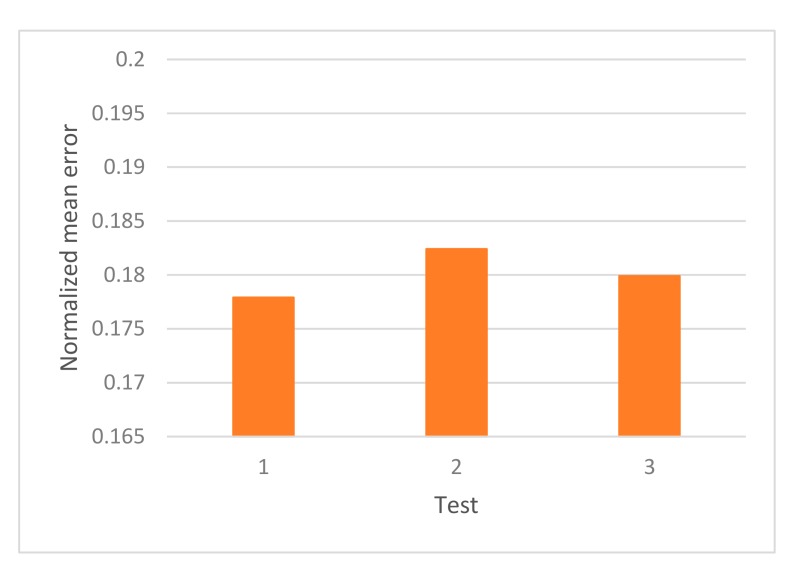
Normalized mean error over the multilayer tests from Table 1.

Considering the multilayer tests, also normalized mean error was used as measure. The results over the multilayer test are 17.79% of error for test 1, 18.24% of error for test 2 and 17.99% of error for test 3, as can be seen in Figure 8.

In this case, the turbulence was stronger and more complex, with the turbulent layers detailed in Table 1; in this case, it was tested, in particular, the capability of the network to generalize. The errors are, on average, slightly bigger than the obtained from the $r_{0}$ tests, although it is limited around 18% of error.

## 4. Discussion

In this work, two different types of results are presented. The first results from the network trained with several sections of the solar image gave a very high mean error, which prevents its use in its actual state as a reconstructor algorithm. Despite the high error, the network response is expected to improve with the use of several fragments of the solar image for the training procedure, refining the training procedure or the simulation specifications. These changes will be taken into account in future works.

The use of one frame of the solar image in the training of the reconstructor provides better results, and consequently, this work focuses in on this case. The development of proto-HELIOS is encouraged from the results obtained in the previous trials, as the network seemed to learn. Selecting just one frame, allows the network to focus more in the slight changes that the turbulence causes in the SH images. This has a drawback, implying that an individual network should be trained for each specific frame of the solar image. However, the presented topology and training procedure showed better results; considering that the cases varied in $r_{0}$, an average error of 14.61% was achieved, which is a better performance than previous approaches [56] where errors of about 20% were reached.

Considering the error behavior when changing $r_{0}$, it has the highest value of error of 31.81%, achieved for the lowest $r_{0}$ value, which is the most turbulent case. The error decreases as $r_{0}$ increases until 14 cm, from where the error value starts raising very slowly. The fact that the lowest error value is found around 12 cm of $r_{0}$ is expectable since the training set is simulated in that $r_{0}$. Although higher $r_{0}$ should mean less turbulent scenarios, the network is not trained specifically in that situation, being the reason for the error to not continue decreasing.

Moreover, the network is capable to obtain less than a 20% of error in multilayer scenarios, where it has not been trained, resulting in an encouraging achievement.

Although the presented results seem reasonable for the complexity of the considered problem, and achieves better results than previous approaches, there are several issues that influence the error and should be attended in future works for developing a full and competitive version of the reconstruction algorithm. Despite the value of the error, the network does learn, thus it is expected that in future works, the use of several fragments of the solar image for the training is feasible, through refining the training procedure or the simulations specifications.

From the simulation point of view, correlations could be tuned up to achieve better precision, as well as some other situations influences the simulations. As solar implies a wide field of view, antialiasing issues should be considered; the SCAO configuration provides the best correction in only one direction in space, but due to the large image of the sun, the corrections are not valid for all angular distances. Additionally, SCAO works on a closed loop, where the limited number of DM actuators makes a constrain in the feedforward simulations. Addressing all these issues could potentially improve the quality of the simulated data, giving a more realistic and reliable training for the network.

For the network, using only fixed images makes it feasible to be used as a reconstruction technique; however, it is preferable to have a network that corrects the turbulence for any section of the sun, to avoid re-training or tuning depending on the case of study.

## 5. Conclusions

The work presented in this paper demonstrates the potential that a neural network has to learn from the atmospheric turbulence effects in solar images and correct them. Although a reconstruction technique for all the regions of the sun is still not reliable, good results have been found when restricting to a fixed section of the sun. At present, the tomographic reconstruction approach with convolutional neural networks is an open line of investigation. Particularly, the reconstruction technique presented, proto-HELIOS, achieves an average 85.39% of precision in the reconstruction, over the test varying the turbulent profiles.

Future work is aimed to a general reconstruction algorithm for solar SCAO, which could be achieved when developed with further improvements in the training procedure. Tuning the simulations and more training capability stands as possible improvements. Other configurations should be considered as well, as MCAO for solar.

## Figures and Tables

**Table 1:** Properties of the parameters for the turbulence in the three multilayer tests.

Test	1	2	3
$r_{0}$ *(m)*	0.085	0.16	0.12
*Turbulence heights (m)*	[0, 6500, 10,000, 15,500]	[0, 4000, 10,000, 15,500]	[0, 6500, 10,000, 15,500]
*Relative strength*	[0.8, 0.05, 0.1, 0.05]	[0.65, 0.15, 0.1, 0.1]	[0.45, 0.15, 0.3, 0.1]
*Wind speed*	[10, 15, 17.5, 25]	[7.5, 12.5, 15, 20]	[7.5, 12.5, 15, 20]
*Wind direction*	[0, 330, 135, 240]	[0, 330, 135, 240]	[0, 330, 135, 240]
